# Tackling the Pharmaceutical Frontier: Regulation of Cannabinoid-Based Medicines in Postwar Japan

**DOI:** 10.1089/can.2015.0011

**Published:** 2016-01-01

**Authors:** Tempei Miyaji, Michiyuki Nagasawa, Takuhiro Yamaguchi, Kiichiro Tsutani

**Affiliations:** ^1^ Department of Clinical Trial Data Management, Graduate School of Medicine, The University of Tokyo, Tokyo, Japan.; ^2^ Department of Pharmaceutical Regulatory Science, Graduate School of Pharmaceutical Sciences, The University of Tokyo, Tokyo, Japan.; ^3^ Division of Biostatistics, Tohoku University Graduate School of Medicine, Sendai, Japan.; ^4^ Graduate School of Pharmaceutical Sciences, The University of Tokyo, Tokyo, Japan.

**Keywords:** regulation, medical marijuana, pharmaceuticals, phytocannabinoids, valley of death

## Abstract

**Background:** The valley of death, which refers to the gap due to the lack of support for the translation of basic research to related clinical studies, exists in the field of translational cannabinoid research in Japan owing to regulations. Article 4 of the Cannabis Control Act (CCA) of 1948 prohibits the use of *Cannabis*-based medicines.

**Objectives:** This study aimed to explore the history of the establishment of regulations on the medical use of *Cannabis*-based medicines and discuss the current status of cannabinoid research and its regulation in Japan.

**Methods:** We conducted a literature review of nationally archived official documents from the end of World War II in 1945 to 1948, which is the year the CCA was established. The documents were examined, specifically focusing on the sequence of events.

**Results:** We found three memoranda related to the establishment of the CCA. The establishment of law on controlling narcotics was instructed by the general headquarters (GHQ)/Supreme Commander for the Allied Powers (SCAP) during the period of occupation after World War II. However, the Japanese Government decided to regulate *Cannabis* separately from other narcotics. Item (ii) of article 4 in the CCA, which prohibits medical application of *Cannabis*, was included to protect farmers growing *Cannabis* for the hemp content.

**Conclusion:** Current Japanese regulations prohibiting clinical research in phytocannabinoids were instituted during the postwar era of World War II. Scientific discoveries have advanced cannabinoid research and have led positive reforms of the regulation of *Cannabis* in other countries. Therefore, there is ample motivation and opportunity for Japanese stakeholders to revise article 4 of the CCA for the benefit of patients.

## Introduction

Over 200 research studies on cannabinoids, including the endocannabinoid system, have been funded by the Grants-in-Aid for Scientific Research of the Ministry of Education, Culture, Sports, Science and Technology (MEXT) of Japan from 1897 to 2014.^1^ For example, the endocannabinoid, 2-arachidonoylglycerol, was discovered by the Japanese researchers Sugiura et al.^2^ simultaneously with Mechoulam et al.^3^ in Israel in 1995. Furthermore, more than 10 of these funded studies were intended to innovate drug development or the clinical application of cannabinoids.^1^ However, the valley of death, which refers to the gap due to the lack of funding and other support for related clinical studies, exists in cannabinoid translational research owing to Japanese regulations. As a result, clinical studies on phytocannabinoids have not been conducted since 1948. In contrast, a number of clinical studies conducted on cannabinoids, including phytocannabinoids, internationally in other countries have already demonstrated their various therapeutic effects in a wide range of common symptoms and rare diseases such as pain, nausea and vomiting, anorexia, depression, epilepsy, and multiple sclerosis by clinical trials.^4^

**FIG. 1.**
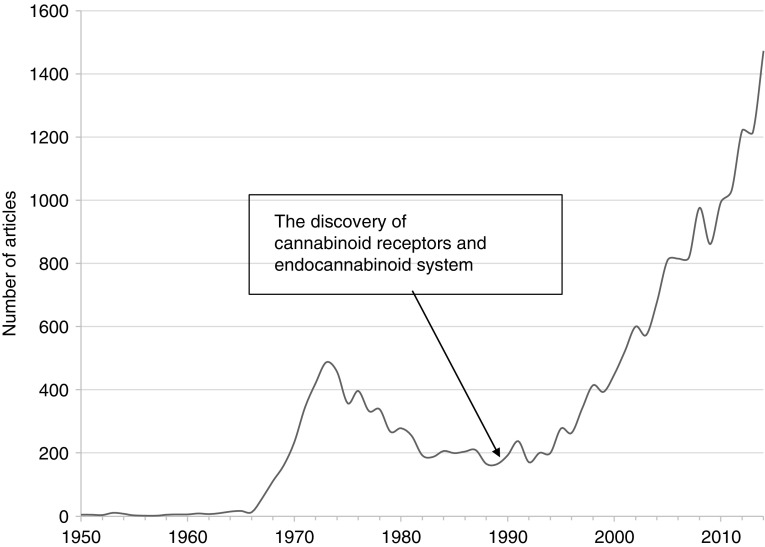
Number of articles related to cannabis or cannabinoids on Pubmed.

Figure 1 shows relevant articles on *Cannabis* or cannabinoids based on their publication year from 1950 to 2014 that are listed in PubMed. Pharmaceutical and medical research on cannabinoids has accelerated rapidly since the discovery of the endocannabinoid system in the human body in the early 1990s.^5^ The development and approval of cannabinoid-based medicine have occurred in European countries and the United States.^6,7^ Moreover, the medical use of *Cannabis* has been authorized under state laws in 23 states and the District of Columbia in the United States as of 2015, including through the Compassionate Use Act.^8^ Furthermore, a major nonpsychoactive cannabinoid, cannabidiol, has been registered by the US Food and Drug Administration and the European Medicines Agency as an orphan drug for the treatment of Dravet syndrome.^9,10^

Although the regulations controlling cannabinoid-based medicine have been positively reformed in other countries, the medical use of *Cannabis* or its components is still strictly prohibited in Japan, and the research in this area is limited to nonclinical studies. Items (ii)–(iii) of paragraph (1) of article 4 in the Cannabis Control Act (CCA, *Taimatorishimarihou*, 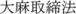)^11^ prohibit the medical use of *Cannabis* with no exceptions for compassionate use or clinical trials. Given below is a quote from related articles of the current version of the CCA (Enactment: Act No. 124 of 1948; Item (iii) was added: Act No. 108 of 1968; Item (iv) was added: Act No. 33 of 1990; Last revision: Act No. 160 of 1999).^12^

***Article 1*** *The term “Cannabis” as used in this Act means the cannabis plant (Cannabis Sativa L.) and its products, provided, however, that the grown stalk of the cannabis plant and its products (excluding resin.) and the seed of cannabis plant and its products are excluded.*

***Article 4 (1)*** *It is prohibited for any person to commit the following acts:*

(i) Importing or exporting Cannabis (excluding cases where the Cannabis Researcher receives authorization from the Minister of Health, Labour and Welfare and imports or exports Cannabis.);
(ii) Treatment with medicines manufactured from Cannabis or distributing it for treatment;
(iii) Receiving treatment with medicines manufactured from Cannabis;
(iv) Advertising Cannabis, except the cases where it is advertised in newspapers or magazines for persons related to pharmaceuticals, etc., (meaning persons related to pharmaceuticals or persons who are engaged in natural science research. The same applies hereinafter in this item.) with articles concerning medical affairs, pharmaceutical affairs, or natural sciences and in other cases where it is advertised mainly to persons related to pharmaceuticals, etc.

The aim of this study is to explore the history of the establishment of regulations on the medical use of *Cannabis*-based medicines and to further discuss the current status of cannabinoid research and its control in Japan.

## Methods

We conducted a thorough literature review of nationally archived official documents from the end of World War II in 1945 to 1948 when the CCA was established. The data were electronically collected by searching PubMed, CiNii books, and the database of the Ichushi (Japan Medical Abstract Society) Web. In addition, the Supreme Commander for the Allied Powers (SCAP) Instruction Note (SCAPIN) was manually searched at the Constitutional Government Reference Room, and the Proceedings of the Diet of the Japanese Government were also electronically searched using the search system of the National Diet Library of Japan.^13^

The collected documents were examined, specifically focusing on information related to the sequence of the events leading to the establishment of the regulations. No official English translation of the Diet Records was found and therefore we translated them in accordance with the Standard Legal Term Dictionary, version 10.^14^ In this study, we defined *Cannabis*-based medicine as medicinal products manufactured from *Cannabis* plants that contain natural phytocannabinoids. Additionally, cannabinoid-based medicine is defined as the medicinal product that includes all ligands of the cannabinoid receptors and related compounds.^4^

## Results

### General headquarters memoranda regulating narcotics

After the end of World War II, Japan was under allied occupation until the Treaty of San Francisco was enforced in 1952. There were 2204 memoranda or instructions in the so-called SCAPIN issued from the general headquarters (GHQ)/SCAP to the Japanese Government to order various kinds of administrative actions or basic measures.^15^ We discovered three memoranda relating to the establishment of the CCA, which were the SCAPIN 130, 644, and 4053-A.

In SCAPIN 130 entitled “Control of Narcotic Products and Records in Japan,” which was instructed on October 12, 1945, the planting and cultivation of narcotic seeds or plants (Supplementary Appendix S1)^16^ as well as importation of related products were prohibited. In paragraph 6, marijuana (*Cannabis sativa* L.) was defined as a narcotic along with opium, cocaine, morphine, and heroin. In addition, in section C of the same paragraph, physicians and apothecaries were included as subjects in this memorandum.

On January 22, 1946, SCAPIN 644 entitled “Establishment of an Effective System for Narcotic Control in Japan” was instructed to the Japanese Government (Supplementary Appendix S2)^17^ and ordered the enactment of the law for narcotic control. Furthermore, in SCAPIN 130, the importation of narcotic products was allowed with the authorization of SCAP, and this memorandum expressed nine requirements for dealing in narcotics, including registration, licensing, and reporting.

On June 28, 1947, SCAPIN 4053-A, which is the amended SCAPIN 130, was instructed (Supplementary Appendix S3).^15^ Although in SCAPIN 130 the importation and production of narcotics were thoroughly prohibited, paragraph 2 of this memorandum states that “*permit the manufacture of finished medicinal narcotic drugs as required for the medical needs of the Japanese people.*” Although the instructions of the memorandum limited the manufacture, importation, and exportation of narcotics, the medical use of narcotic drugs manufactured from *Cannabis* was not prohibited by the GHQ/SCAP and therefore medical applications were an exception as of June 1947.

### Debate of the Japanese Diet Committee

After receiving these memoranda, the Welfare Committee of the Diet held a preparatory meeting to discuss enacting the law of narcotic control. Table 1 chronologically presents the events related to the establishment of the CCA. Before the medicinal aspects of *Cannabis* were mentioned in the Diet memorandum, there had been concerns raised by farmers because it has been inseparably linked to Japanese spirituality and lifestyle from ancient times. While the origins of hemp are not entirely clear, hemp was most certainly first imported to the island of Kyushu from China through Korea in the Neolithic Jomon Period (10,000—300 BC).^18^ Cannabis has been used as textiles, foods, and medicines, as well as in the Shinto rituals. *Cannabis* was already subject to control by the revised International Opium Convention adopted by the Second Opium Conference of 1925, which Japan ratified in 1928 and implemented as the Narcotics Control Regulation in 1930. However, the use of *Cannabis* for medical and scientific purposes was exempted by the convention.^19^ There was a huge demand for hemp fiber from *Cannabis* for national restoration during the postwar era^20^ and therefore there were concerns regarding placing restrictions on its cultivation among farmers.

**Table 1.** **Events Related to the Establishment of the Cannabis Control Act**

Date	Event
12 October, 1945	SCAPIN 130 “Control of Narcotic Products and Records in Japan” instructed
22 January, 1946	SCAPIN 644 “Establishment of an effective System for Narcotic Control in Japan” instructed
28 June, 1947	SCAPIN 4053-A “Authorization to Manufacture Narcotics in Japan” instructed
12, 15–17 June, 1948	Deliberation at The Welfare Committee of the House of Representatives
19 June, 1948	Deliberation at The House of Representatives
24–25 June, 1948	Deliberation at The Welfare Committee of the House of Councilors
28 June, 1948	Deliberation at The House of Councilors
10 July, 1948	CCA promulgated and enforced
1 March, 1951	Three commodities of Cannabis-based medicines were deleted from the sixth edition of Japanese Pharmacopoeia
17 March, 1953	Cannabis seeds were excluded from the control
21 June, 1963	Item (iii) was added in article 4 of CCA
19 June, 1990	Item (iv) was added in article 4 of CCA

CCA, Cannabis Control Act; SCAPIN, Supreme Commander for the Allied Powers Instruction Note.

Given below is the statement of Mr. Kihachiro Honma, a member of the Petition Committee of the House of Representatives (HoR), June 3, 1947.^21^

“*Cannabis has a very wide range of use, thus it is a very important substance. However, the cultivation of Cannabis plant was prohibited due to the narcotic effects of the active ingredient in Cannabis as mentioned above. I, as a staff of Ministry of Agriculture and Forestry, have supplicated to GHQ/SCAP for the cultivation to continue so that farmers growing hemp fiber will not be damaged economically and the present imbalance of supply and demand on hemp products will be dissolved.*”

Therefore, considering the impact of the prohibitory laws on agricultural cash crop cultivation, a member of the Welfare Committee of the HoR proposed a separation of the law of *Cannabis* and that of other narcotics, which would allow farmers to cultivate hemp with fewer restrictions compared with plants that are raw material for narcotics. Given below is the statement of the Minister of State, Mr. Giichi Takeda, presented at the Welfare Committee of the HoR on June 12, 1948.^21^

“*I would now like to address the topic of conversation, the draft of CCA. Because the resin contained in the Cannabis plant has narcotic effects, Cannabis has been cracked down as a narcotic. Most people who are cultivating Cannabis plant are in agriculture, and those who will be subjected to the license system by the draft of Narcotics Control Act (NCA) are in vastly different occupations, such as doctors, dentists, and pharmacists, etc.*, *making the draft of CCA a good balance considering this situation and perfecting enforcement. This is the reason why the draft was submitted separately from the draft of NCA.*”

Following the passing of the CCA draft by the HoR, further discussions on the use of *Cannabis*-based medicines were conducted in the House of Councilors (HoC). Item (ii) of article 4 was intended to control products that had already been distributed in the market and given below is the discussion that ensued between two members of the Welfare Committee of the HoC on June 25, 1948^21^;

Mr. Ryuen Kusaba asked,

“*Article 4 mentions the term “treatment,” which I heard includes the prescriptions of a drug manufactured from Cannabis. If the basis of this Act is that all drugs manufactured from Cannabis are to be prohibited, there will be no treatment with medicines manufactured from Cannabis. Then, the term “treatment” is unnecessary, leading us to question why item (ii) is submitted with such contradiction.*”

Mr. Katsuji Kuge (a staff of the Ministry of Health and Welfare) subsequently responded,

“*I will take this question. We think that previously manufactured Cannabis-based medicines still remain in Japan. Regarding such meaning, this item has been added in article 4.*”

However, the proceedings of the Diet discussion on the medical use of *Cannabis* only involved the above conversations, and the draft of CCA was subsequently passed by the Welfare Committee of the HoC on June 25, 1948, and by the HoC itself on June 28, 1948.

### Establishment of CCA

On July 10, 1948, the CCA was promulgated and enforced. Three commodities concerning *Cannabis*-based medicines were listed in the Japanese Pharmacopoeia, namely *Cannabis indica, Extractum Cannabis indica* (1st edition, 1886), and *Tinctura Cannabis indicae* (4th edition, 1920); however, these items were deleted in the review process of the sixth edition (1951).^19^ Since then and to date, both the clinical use of *Cannabis*-based medicines and related clinical studies have been prohibited in Japan.

## Discussion

### Separation of CCA and NCA

The establishment of the law controlling *Cannabis* was initially led by the GHQ/SCAP in the postwar period following World War II and it was instructed along with other narcotic control measures. In describing the related SCAPIN, the GHQ/SCAP exempted the use of narcotics for the medical needs of Japanese people from prohibition by SCAPIN 4053-A. We were unable to locate records of the negotiations between the GHQ/SCAP and the Japanese Government in this present study. However, from the available information in the Diet Records, the Japanese Government appeared to have made a proposal for the separation of the CCA and the NCA to the GHQ/SCAP to protect farmers and enable them to continue to engage in hemp agricultural production.

Then, why was the separation of law necessary to protect the farmers? Table 2 shows the relationship of the control of cultivation and medical use of narcotics with the CCA^11^ and the NCA.^22^ The CCA allows the cultivation of *Cannabis* with a cultivator license, but not medical use. On the other hand, the NCA allows medical use of narcotics with a license, but not cultivation. Cultivation of *Cannabis* has numerous purposes such as for food, fiber, and medicine, whereas other narcotic plants are grown mainly as sources of drugs. If CCA had allowed not only cultivation but also medical use, its enforcement would have been difficult in terms of controlling the medical use because it was laborious to distinguish which *Cannabis* farms for which uses especially in the confused period just after the end of the war. Therefore, in our opinion, the Japanese Government has stifled the medical use of *Cannabis* to continue allowing its cultivation by hemp farmers and, thereby, has protected the industry. Indeed, the current version of the CCA only regulates the handling of *Cannabis* leaves and spikes, which contain psychoactive substances, while the seeds and stem, which are the resource of food and fiber, were excluded from the control. When the laws were enacted in 1948, *Cannabis* seeds were also subject to the CCA, but were excluded from the control by the third revision of March 17, 1953. This amendment was also intended to simplify the control and facilitate the ease of cultivation.

**Table 2.** **Relationship Between Cultivation and Medical Use of Cannabis and Other Narcotics**

	Applicable legislation	Cultivation	Medical use
Cannabis	CCA	Allowed with license	Prohibited
Narcotics^a^	NCA	Prohibited^b^	Allowed with license

^a^ Excluding Cannabis.

^b^ Only a research license holder can cultivate with authorization of Ministry of Health, Labour and Welfare for study purpose.

### Problem associated with article 4 of the CCA

As mentioned previously, although the CCA prohibits the medical use of *Cannabis* and its extracted products in article 4, it does not regulate the mature stalks, seeds, and related products. It only regulates the leaves and spikes of *Cannabis*. Therefore, *Cannabis*-based medicines, which are extracted from the leaves and spikes, such as the phytocannabinoid formulation, nabiximols (Sativex^®^),^6^ are subject to the CCA and not approved for use in humans. On the other hand, dronabinol (Marinol^®^)^7^ or nabilone (Cesamet^®^),^23^ constituted from synthetic tetrahydrocannabinol and not *Cannabis*, is not subject to the CCA and therefore may possibly be investigated in clinical trials. Indeed, a clinical trial of dronabinol was planned according to the research report of the Ministry of Health, Labour and Welfare (MHLW)-funded project,^24^ but was not initiated. Moreover, rimonabant (Acomplia^®^), the synthetic cannabinoid receptor 1 antagonist, was clinically investigated as a weight-loss drug in Japan; however, the trial was terminated due to evidence of an unfavorable neuropsychiatric adverse reaction in other trials in 2009.^25,26^ From a scientific viewpoint, there is no plausible reason to allow the clinical investigation of synthetic cannabinoids, but not phytocannabinoids.

In 1948, cannabinoids and the endocannabinoid system had not yet been discovered, and the therapeutic properties of *Cannabis* were not well investigated or provided with a scientific basis. However, the scientific discovery process of the cannabinoids has been accelerated, which includes investigation of efficacy and safety profiles in clinical trials and this has initiated global approval of phytocannabinoid-based medicines. Therefore, there is no current plausible reason for the regulatory prohibition of investigations of phytocannabinoid-based medicines in Japan. The evidence of the existence of an endocannabinoid system implies that phytocannabinoids are potentially useful substances for medical applications. The argument that the clinical investigation of synthetic cannabinoids, which is permitted under the current regulation, is sufficient for pharmacological assessments in drug development may be proffered. However, synthetic cannabinoid medicines usually consist of a single agent, while phytocannabinoid-based medicines and medicinal *Cannabis* contain various types of cannabinoids as well as terpenoids that might induce the entourage effects to complementary therapeutic activities.^27^ Therefore, by focusing on potential phytocannabinoid–terpenoid synergistic effects, pharmaceutical development of phytocannabinoids could advance the pharmaceutical frontier of novel drug discovery.

Ultimately, reforming the regulations would be an ethical response to patients who have been appealing for the access to treatment of their diseases with possible phytocannabinoid-based medicines. The regulations need to be positively revised to meet the medical requirements and rights of the patients. It is noteworthy that in 1999, a patient advocacy group commenced appeals for the revision of the CCA in Japan. However, as a prerequisite for the revision, stakeholders need to cooperate with legal professionals to establish a new comprehensive regulatory system to strictly guide the appropriate use of cannabinoid-based medicines, including medical *Cannabis*. It is important to prevent the risk of any abuses or evasions of law by learning lessons from the precedents of other countries and developing rigid schemes to control cannabinoid-based medicine, especially medical *Cannabis*. Legislating a compassionate use program might be one of the choices to improve the current situation without revising article 4 of the CCA. Moreover, the authors are not in a position to deregulate the recreational use of *Cannabis*.

### Article 4 of the CCA awaits appropriate revision

The demonstration of the existence of the endocannabinoid system in the human body implies that cannabinoids are essential substances and they can be positively applied as therapies for various symptoms and diseases. There are over 200 basic research studies on cannabinoids that have been supported by the Grants-in-Aid for Scientific Research from the MEXT, and more than 10 of the investigated topics are intended to innovate the drug development or clinical application of Cannabinoids.^1^ Unfortunately, these basic research findings on phytocannabinoids cannot be translated to clinical studies due to the current regulation in Japan. These regulatory prohibitions have contributed to creating the valley of death, which may be difficult to overcome without revising article 4 of the CCA or creating new regulations such as a compassionate use program. Phytocannabinoids have been neglected as potentially beneficial therapeutic agents in clinical research for almost 70 years in Japan.

Fortunately, Japan recently stepped up efforts to institutionalize changes to foster the Japanese medical research environment. The Independent Administrative Agency of Japan, Agency for Medical Research and Development (AMED), which commenced activities in April 2015,^28^ was established based on new laws called the Acts on Promotion of Healthcare and Medical Strategies and the Establishment of AMED. AMED aims to promote integrated research and development in the field of medicine from basic research to clinical trials by consolidating budgets for research expenses, which were previously allocated to several ministries such as the MEXT and MHLW for basic and clinical research, respectively.^28^ Japan is currently reforming the medical research environment to resolve drug development lapses and promote innovate research areas, including regenerative medicine and oncology. Thus, the nation now has a conducive environment and the opportunity to positively revise the regulation on cannabinoids. The government, academia, industries, and medical and legal professionals need to come together cooperatively and address these issues with all seriousness and a view to resolving them.

## Conclusion

Currently, clinical research on phytocannabinoids is prohibited in Japan based on regulations that were instituted during the postwar period of World War II, which have contributed to the valley of death in cannabinoid translational research. These prohibitive regulations have been an undesired obstacle to the phytopharmacological development of *Cannabis* for almost 70 years. Scientific discoveries in cannabinoid research have accelerated our understanding of the potential benefits of these agents. Consequently, there have been positive reforms of the regulations governing *Cannabis* globally, and Japanese regulatory bodies are now faced with the window of opportunity to revise article 4 of the CCA.

## Supplementary Material

Supplemental data

Supplemental data

Supplemental data

## Abbreviations Used

AMED: Japan Agency for Medical Research and Development
CCA: Cannabis Control Act
GHQ: general headquarters
HoC: House of Councilors
HoR: House of Representatives
MEXT: Ministry of Education, Culture, Sports, Science and Technology
MHLW: Ministry of Health, Labour and Welfare
NCA: Narcotics Control Act
SCAP: Supreme Commander for the Allied Powers
