# Supplementary material for: Tackling the Pharmaceutical Frontier: Regulation of Cannabinoid-Based Medicines in Postwar Japan
Source: Cannabis Cannabinoid Res. 2016 Jan 1;1(1):31–7. doi: 10.1089/can.2015.0011 (PMC5576599; doi:10.1089/can.2015.0011)
Supplement: Supplemental data [file Supp_Appendix1.pdf]

GENERAL HEADQUARTERS
SUPREME COMMANDER FOR THE ALLIED POWERS

APO 500
12 October 1945

AG 441.1 (12 Oct 45)PH
(SCAPIN - 130)

MEMORANDUM FOR: IMPERIAL JAPANESE GOVERNMENT.

THROUGH : Central Liaison Office, Tokyo.

SUBJECT : Control of Narcotic Products and Records in Japan.

1. The planting, cultivation, or growth of narcotic seeds or plants is prohibited. All narcotic seeds or plants which are now planted, being cultivated or grown will be destroyed immediately. The quantity so destroyed, date and method of destruction, location and ownership of the fields or areas will be reported to the Supreme Commander for the Allied Powers within thirty days.

2. The importation of narcotics by any person is prohibited except as authorized by the Supreme Commander for the Allied Powers.

3. The exportation or manufacture of narcotics is prohibited.

4. All stocks of crude, semi-processed or smoking opium; crude or semi-processed cocaine; heroin and marijuana (Cannabis Sativa L) are hereby frozen and the removal, destruction, use or sale thereof or of any books or records thereof is prohibited except as authorized by the Supreme Commander for the Allied Powers.

5. All existing records of narcotic transactions in narcotics shall be maintained.

6. Definitions:

a. Narcotic or Narcotics shall include Opium, Cocaine, Morphine, Heroin, Marijuana (Cannabis Sativa L), their seeds and plants, and every substance in any way derived therefrom, or any mixture or preparation thereof.

b. Heroin shall include any derivative, compound, salt, mixture, or preparation thereof.

BASIC: Memo to Imperial Japanese Government, file AG 441.1 (12 Oct 45)PH, subject: "Control of Narcotic Products and Records in Japan", dtd 12 October 1945.

c. Person shall include physicians, dealers, apothecaries, government monoplies, and all other individuals, depositories, partnerships, corporations, unincorporated business firms or associations, and all responsible members thereof.

FOR THE SUPREME COMMANDER:

/s/ H. W. Allen,
/t/ H. W. ALLEN,
Colonel, A. G. D.,
Asst Adjutant General.
