# Supplementary material for: Tackling the Pharmaceutical Frontier: Regulation of Cannabinoid-Based Medicines in Postwar Japan
Source: Cannabis Cannabinoid Res. 2016 Jan 1;1(1):31–7. doi: 10.1089/can.2015.0011 (PMC5576599; doi:10.1089/can.2015.0011)
Supplement: Supplemental data [file Supp_Appendix2.pdf]

GENERAL HEADQUARTERS
SUPREME COMMANDER FOR THE ALLIED POWERS

AG 441.1 (22 Jan 46)PH
(SCAPIN - 644)

22 January 1946

MEMORANDUM TO: IMPERIAL JAPANESE GOVERNMENT.

THROUGH : Central Liaison Office, Tokyo.

SUBJECT : Establishment of an Effective System for Narcotic Control in Japan.

1. You are directed to enact legislation embodying the following provisions for the establishment of an effective system for narcotic control in Japan:

a. All persons lawfully entitled to deal in narcotics will be properly registered, licensed, and classified.

b. Dealers will be required to re-register annually.

c. Dealers will be required to submit an inventory of narcotic stocks on hand when registering or re-registering.

d. Dealers will be required to submit periodical reports of narcotic transactions and stocks on hand to the Imperial Japanese Government.

e. All transactions in narcotics will be in or from original stamped packages which you will provide for in your enactment.

f. Limitations of sales by the several classes of registrants will be set forth in your enactment.

g. Sales between registrants will be by means of order forms which you will record and provide in blank to registrants.

h. Except as provided in your enactment any person who is not registered, licensed and classified will be prohibited from having narcotics in his possession.

i. Severe penalties will be provided for any person who violates the provisions of your enactment.

2. You will submit a copy of the law in English to this Headquarters prior to enactment.

FOR THE SUPREME COMMANDER:

H. W. ALLEN,
Colonel, AGD,
Asst. Adjutant General
