# Supplementary material for: Tackling the Pharmaceutical Frontier: Regulation of Cannabinoid-Based Medicines in Postwar Japan
Source: Cannabis Cannabinoid Res. 2016 Jan 1;1(1):31–7. doi: 10.1089/can.2015.0011 (PMC5576599; doi:10.1089/can.2015.0011)
Supplement: Supplemental data [file Supp_Appendix3.pdf]

主管 經濟部 厚生省[illegible]課 医療用麻薬製剤品の製造許可に関する件

GENERAL HEADQUARTERS
SUPREME COMMANDER FOR THE ALLIED POWERS

AG 441.1 (28June 47)PH
(SCAPIN 4053 -A)

APO 500
28 June 1947

MEMORANDUM FOR: JAPANESE GOVERNMENT

THROUGH: Central Liaison Office, Tokyo

SUBJECT: Authorization to Manufacture Narcotics in Japan

1. Reference is made to Memorandum number AG 441.1 (12 Oct 45)PH, 12 October 1945, subject: "Control of Narcotic Products and Records in Japan".

2. Paragraph 3 of the referenced memorandum is amended, as an interim measure, to permit the manufacture of finished medicinal narcotic drugs as required for the medical needs of the Japanese people.

3. Crude and semi-processed narcotics now held in custody by Occupation Forces shall be received, receipted for, and delivered into the custody of duly licensed manufacturers by the Japanese Government as approved by General Headquarters, Supreme Commander for the Allied Powers.

4. Complete monthly reports of stocks on hand shall be submitted to General Headquarters, Supreme Commander for the Allied Powers, by the Japanese Government no later than the last day of the month following the month for which the report is made.

5. Direct communication with the Narcotic Control Branch, Public Health and Welfare Section, General Headquarters, Supreme Commander for the Allied Powers, is authorized for the entire process including the plan for accomplishing manufacture of the narcotics, transfer of custody, secure storage of stocks, and estimating requirements for the medical needs of the Japanese people.

FOR THE SUPREME COMMANDER:

Received: 30 Jun, 11,00 a.m.
Shukan: EWL 済
Copy: D of GA, DAE, D of Toyaku, PH, Print

R. M. LEVY
Colonel, AGD
Adjutant General

22. 6. 30
